# On Wandering Erysipelas

**Published:** 1831

**Authors:** 


On Wandering Erysipelas. By M. Rennes, M.D. of St. Omer.

Dr. R. has published a paper under the above head in a late number of the Archives, and elucidates the subject by the two following cases.

*Case* 1. A soldier of the 15th regiment, 21 years of age, of sanguineo-lymphatic temperament, had suffered slightly from otitis, succeeded by a discharge from the ear. On the 2d March the discharge suddenly stopped, and on the succeeding day was replaced by erysipelas over the whole of the left side of the face. In this state he entered the Hospital of St. Omer, where Dr. R. is chief physician. The head was heavy, the pulse full and frequent, skin hot, thirst urgent, tongue edged with red, abdomen rather sensible to pressure. Venesection—20 leeches to the epigastrium. 5th. The erysipelas was less vivid, the fever moderated. There was some delirium in the night. Tartrite of antimony and cream of tartar drink, and in the evening sixteen leeches to the neck. 6th. The erysipelas had subsided on the left side of the face; but it now commenced on the opposite side, with a great increase of fever and delirium. *No discharge from the bowel for several days.* The same drink to be continued. In the course of the day the erysipelas extended to the neck and shoulders. Evening, *no evacuation from the bowels.* The pulse is quick and sharp—skin hot and dry.—20 leeches to the neck—two grains of tartar emetic in a lavement. The consequence of this last remedy was *several bilious stools.* The night was passed in *tranquillity,* and next morning the patient was much better in every respect. The fever soon subsided, but the erysipelas continued to spread downwards. Another emetic glyster was exhibited, and produced copious bilious stools. Although the erysipelas continued to spread even to the extremities, the constitutional symptoms were mild, and on the 26th of March the patient was discharged from the hospital.

*Remarks.* It is impossible not to perceive the injurious consequences which flowed in the above case, from the antipathy which our Gallic neighbours bear towards aperient medicine. This man had no evacuation from the bowels for many days, and the erysipelas, as well as the constitutional disturbance, became daily exasperated. Yet no purgative was even dreamt of; and until the two grains of tartrite of antimony were exhibited in lavement, the disease received no check whatever from any thing that was done. The bilious evacuations instantly lowered the constitutional disturbance, and although the cutaneous affection continued to spread, yet the whole of morbid phenomena were greatly mitigated in severity, and ended safely.

*Case* 2. This was a young lady, who became affected with erysipelas of the face, accompanied by constipated bowels, and she underwent the usual routine of leeching, mustard poultices, blistering, and glystering, without the *slightest* relief. On the contrary, the constitutional symptoms rose to such a height that her life was despaired of; yet no aperient medicine was ever prescribed!! At length Nature seems to have effected what the physician did not aim at—the evacuation, "pour la première fois quelques grumeaux de *matieres noiratres.*" This discharge of black and grumous matters, however, was too scanty to produce much effect, and the erysipelas, with all its constitutional symptoms, went on unmitigated for a day or two longer. After a very dangerous state, this lady ultimately emerged into convalescence.

*Remarks.* These two cases are valuable in shewing the necessity of clearing the bowels effectually, and indeed daily, of all vitiated secretions and excretions, during the progress of erysipelas. It is marvellous that the French physicians continue nearly as blind as ever to the benefit of purgation in acute diseases.

